# A Positive Feedback Loop of Profilin-1 and RhoA/ROCK1 Promotes Endothelial Dysfunction and Oxidative Stress

**DOI:** 10.1155/2018/4169575

**Published:** 2018-04-04

**Authors:** Xu Li, Jianjun Liu, Bin Chen, Longhua Fan

**Affiliations:** ^1^Department of Vascular Surgery, Qingpu Branch of Zhongshan Hospital, Fudan University, Shanghai 200032, China; ^2^Institute of Vascular Surgery, Department of Vascular Surgery, Zhongshan Hospital, Fudan University, Shanghai 200032, China

## Abstract

Vascular endothelial dysfunction is considered critical development in the progression of cardiovascular events and is associated with vascular damage and oxidative stress. Previous studies have shown that profilin-1 could be induced by advanced glycation end products (AGEs) and contributes to vascular hyperpermeability; however, the mechanisms are not fully understood. In this study, we sought to assess whether reactive oxygen species (ROS) were involved in profilin-1-mediated RhoA/ROCK1 signaling. Treatment with AGEs significantly induced the expression of profilin-1 and ROS production in human umbilical vein endothelial cells (HUVECs), whereas knockdown of profilin-1 diminished AGE-induced RhoA and ROCK1 activation and ROS production. Moreover, ectopic overexpression of profilin-1 also increased RhoA and ROCK1 activation and ROS production under low AGE concentration. Furthermore, blockage of RhoA/ROCK1 with the inhibitors CT04 and Y27632 significantly attenuated the profilin-1-mediated cell damage and ROS production. Additionally, ROS inhibition resulted in a significant decrease in profilin-1-mediated RhoA/ROCK1 expression, suggesting that the regulation of RhoA/ROCK1 signaling was partly independent of ROS. Taken together, these results suggested that the RhoA/ROCK1 pathway activated by excessive ROS is responsible for profilin-1-mediated endothelial damage.

## 1. Introduction

Endothelial dysfunction is one of the most critical risk factors of diabetes leading to morbidity and is characterized by insulin deficiency or impaired insulin signaling [1, 2]. Increasing amounts of evidence have shown that oxidative stress is associated with the pathogenesis of many diseases, including diabetes [3–5]. Although there has been a significant amount of study into the precise mechanisms of oxidative stress in vascular dysfunction in diabetes [6–8], there is a dearth of effective strategies to decrease the incidence of diabetic vascular disease. More progress in the understanding of endothelial dysfunction at the molecular level is urgently needed in order to develop new therapies for clinical application.

Profilin-1, a member of the profilin family, was first characterized as a small actin-binding protein and is widely distributed in various types of cells [9]. Profilin-1 has been found to be increased in the endothelial cells and macrophages of atherosclerotic lesions in diabetic individuals, and oxidized LDL (oxLDL) was capable of triggering profilin increases [10]. Further investigation has shown that a knockdown of profilin-1 was protective against endothelial dysfunction triggered by oxLDL in cultured aortic endothelial cells [11]. Overexpression of transgenic profilin 1 resulted in the elevation of blood pressure via medicating vascular remodeling [12]. Profilin-1 also plays a crucial role in hypertension-induced artery remodeling through the p38-iNOS-peroxynitrite pathway [13]. Recently, profilin-1 was reported to be involved in endothelial injury induced by AGEs [14]. RhoA/ROCK1 has been reported to downregulate eNOS, which results in impaired endothelial function and vascular relaxation [15–17]. These data suggest that profilin-1 plays a critical role in regulation of oxidative stress and vascular remodeling. However, the exact roles and mechanisms of profilin-1 in endothelial abnormalities and vascular disease remain largely unknown.

In this work, we assume that profilin-1 is induced by AGEs and that AGE-stimulated profilin-1 activation is necessary for ROS production and endothelial injury. The aim of the present study was to clarify the molecular mechanisms of profilin-1 in endothelial injury, with the goal of identifying a potential target for genetic therapy of diabetes-induced arterial remodeling.

## 2. Methods and Materials

### 2.1. Cell Culture and Treatments

Human umbilical vein endothelial cells (HUVECs) were cultured as described previously [18]. Cell-permeative Rho inhibitor C-3 transferase (CT-04), a RhoA inhibitor, was purchased from Cytoskeleton (Denver, CO, USA). Y-27632, Rho kinase inhibitor, and Tiron (4,5-dihydroxy-1,3-benzene disulfonic acid-disodium salt), an ROS scavenger, were purchased from Sigma-Aldrich (St. Louis, MO, USA) and dissolved in DMSO.

### 2.2. Plasmids and Transfection

Human profilin-1 cDNA was subcloned into pCDH-CMV-MCS-EF1-copGFP lentiviral vector (System Biosciences, Mountain View, CA, USA). Lentiviral vectors harboring the profilin-1 expressing plasmid accompanied with the pPACKH1 HIV Lentivector Packaging Kit (System Biosciences) were transfected into HEK-293 T cells according to manufacturer's instructions. HUVECs were infected with profilin-1 in the presence of 4 *μ*g/ml polybrene (Sigma-Aldrich, St. Louis, MO, USA) and selected using a Coulter EPICS Elite ESP flow cytometer (Beckman, Miami, Florida, USA) based on the expression of GFP. For knockdown assays, profilin-1, siRNA targeting profilin-1, and the scrambled nontarget control (NC) were purchased from GenePharma (Shanghai, China) and transfected into HUVECs using HiPerFect transfection reagent (Qiagen, Valencia, CA, USA) according to manufacturer's instructions.

### 2.3. Apoptosis Assay

Apoptosis of cells was determined by flow cytometric analysis using PE Annexin V Apoptosis Detection Kit I (BD Biosciences, Franklin Lakes, NJ, US) as described previously [18].

### 2.4. Cell Migration Assay and Tube Formation Assay

Cell migration potential was assessed by transwell assays as previously reported [18]. For the tube formation assay, after appropriate treatment, approximately 4 × 10^4^ HUVECs (in 400 *μ*l) were added into a 24-well plate, precoated with Matrigel (BD Biosciences), and incubated for 6 h. Random photographs were taken under a light microscope (Olympus, Tokyo, Japan) at 100x magnification to assay for the formation of capillary-like structures.

### 2.5. Intracellular ROS Measurement

Intracellular ROS was measured using 2,7-dichlorodihydrofluorescein diacetate dye (H2DCFDA, Life Technologies) as described previously [19].

### 2.6. Western Blots and RhoA Pull-Down Assay

Western blotting was performed using standard methods as previously reported [19]. Antiprofilin-1 antibody (#3237), anti-ROCK1 (#4035), and anti-p-eNOS antibodies (#9570) were purchased from Cell Signaling Technology (Beverly, MA, USA). Anti-*β*-actin antibody (sc-47778) was obtained from Santa Cruz Biotechnology (Santa Cruz, CA, USA).

RhoA activation was assessed using the RhoA Activation Assay Biochem Kit (Cytoskeleton, Denver, CO, USA) according to previously described methods [18].

### 2.7. Statistical Analysis

The data are presented as the mean ± SD (standard deviation) of at least three independent experiments. Student's *t*-test was used to compare two groups, and one-way analysis of variance (ANOVA) was used to compare multiple variables. *P* < 0.05 was considered as significant.

## 3. Results

### 3.1. AGEs Upregulate Profilin-1 Expression, Endothelial Injury, and ROS Generation in HUVECs

**Figure 1:**
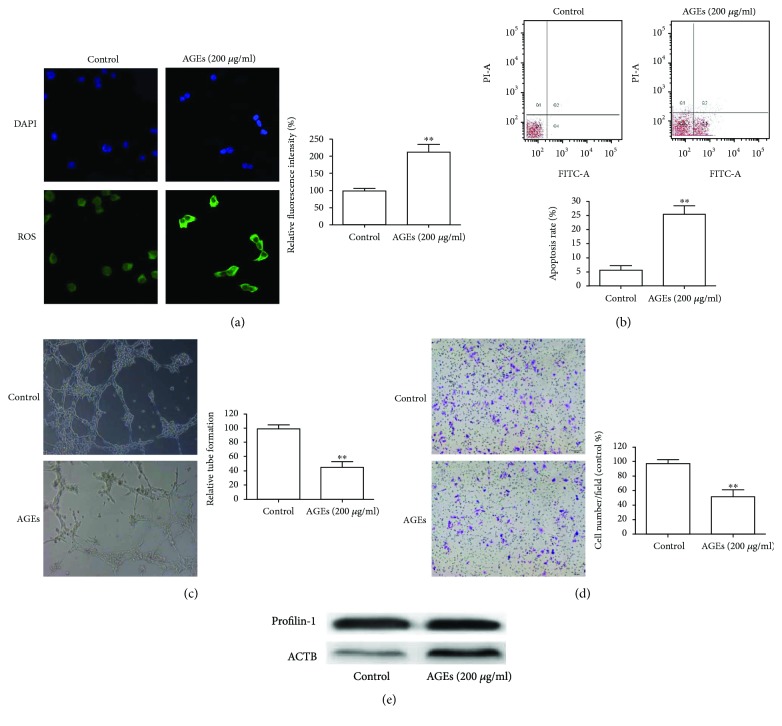
Effects of AGEs on HUVECs and profilin-1 expression for (a) intracellular ROS, (b) apoptosis, (c) tube formation, and (d) migratory abilities were measured in HUVECs treated with 200 *μ*g/ml AGEs for 24 h. (e) Western blotting for profilin-1 in HUVECs after AGE treatment for 24 h. *β*-actin serves as a loading control. ^∗∗^*P* < 0.01.

Previous studies have shown the effects of AGEs on HUVECs [14, 20], but the results were inconsistent. We first confirmed the functions of AGEs on HUVECs. ROS production in HUVECs was significantly increased after a 24 h treatment with 200 *μ*g/ml AGEs (Figure 1(a)). Flow cytometric analysis was performed to assess the effects of AGEs on cell apoptosis. Results indicated that cell apoptosis was significantly increased after AGE treatment (Figure 1(b)). The tube formation assay also showed that treatment with AGEs significantly decreased the ability to form tubes (Figure 1(c)). In addition, the ability of cells to migrate was also significantly hampered after AGE treatment (Figure 1(d)). Western blot analysis revealed that AGEs increased the expression of profilin-1 in HUVECs exposed to AGEs (Figure 1(e)).

### 3.2. Inhibition of Profilin-1 Attenuates AGE-Induced Endothelial Damage and ROS Generation in HUVECs

**Figure 2:**
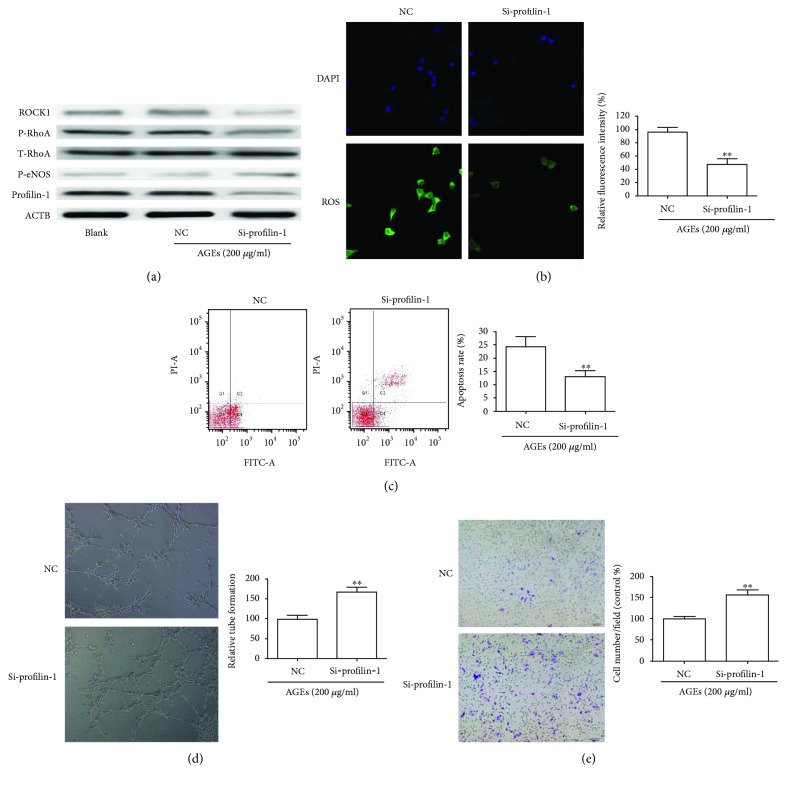
Knockdown of profilin-1 attenuated AGE-induced cell damage and ROS production. HUVECs were transfected with control siRNA (NC) and profilin-1 siRNA (si-profilin-1) for 24 h and then stimulated with AGEs for 24 h. (a) Western blotting demonstrated the expression of profilin-1, T-RhoA, p-RhoA, p-eNOS, and ROCK1. (b) Intracellular ROS, (c) apoptosis, (d) tube formation, and (e) migratory abilities were detected as indicated. ^∗∗^*P* < 0.01.

Profilin-1 has been shown to be associated with endothelial abnormalities and ROS production in HUVECs [14]. We examined whether profilin-1 was involved in the activation of ROS production by AGEs. Profilin-1 expression was significantly reduced in cells transfected with profilin-1 siRNA compared with blank (not transfected) or NC cells (Figure 2(a)). Inhibition of profilin-1 in HUVECs resulted in a drastic decrease of ROS (Figure 2(b)). Flow cytometry showed that knockdown of profilin-1 reversed the apoptotic effects caused by AGEs (Figure 2(c)). The average number of tubular structures was increased when profilin-1 was silenced (Figure 2(d)). Migration assays showed that HUVECs transfected with profilin-1 siRNA displayed a notable increase when compared with NC (Figure 2(e)), followed by a decrease in the expression of ROCK1 and p-RhoA, and an increase in the expression of p-eNOS (Figure 2(a)).

### 3.3. Overexpression of Profilin-1 Promotes Endothelial Damage and ROS Generation under AGE Treatment

**Figure 3:**
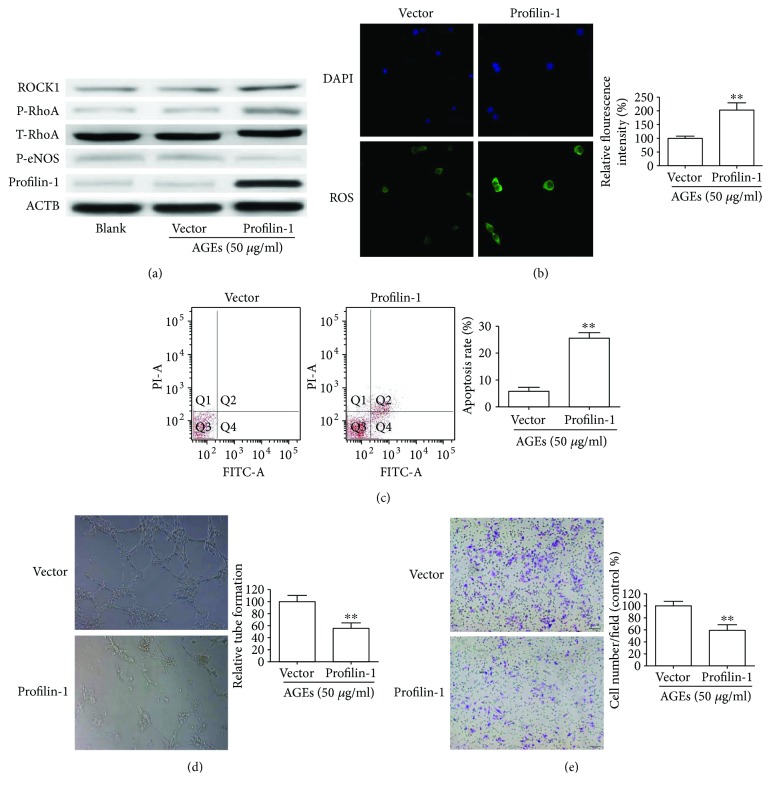
Profilin-1 aggravated cell damage and ROS production under AGEs. Stable overexpression of profilin-1 in HUVECs was treated with 50 *μ*g/ml AGEs for 24 h. (a) The effects of profilin-1 on the expression of T-RhoA, p-RhoA, p-eNOS, and ROCK1 were detected by Western blotting. (b) Intracellular ROS, (c) apoptosis, (d) tube formation, and (e) migratory abilities were detected in HUVECs stimulated with 50 *μ*g/ml AGEs for 24 h. ^∗∗^*P* < 0.01.

To further explore whether profilin-1 was required for AGE-induced endothelial damage and ROS production, we investigated the effects of overexpression of profilin-1 on the biological behavior of HUVECs. HUVECs were transduced with profilin-1, and expression was confirmed by Western blot (Figure 3(a)). Compared with vector-only cells, HUVECs transduced with profilin-1 had significantly increased ROS production based on DCFDA assays (Figure 3(b)). Flow cytometry analysis of HUVECs showed that overexpression of profilin-1 induced apoptosis when compared with the vector-only cells (Figure 3(c)). Tube formation assays indicated that overexpression of profilin-1 could lead to a decrease in tube formation (Figure 3(d)). Moreover, transwell assays showed that increased profilin-1 expression inhibited HUVEC migration (Figure 3(e)). All the above results were obtained from HUVEC cells treated with 50 *μ*g/ml AGEs. However, there was no significant change in HUVECs under normal state (data not shown). Consistent with cell data, Western blot analysis demonstrated that profilin-1 increased the expression of ROCK1 and p-RhoA and decreased the expression of p-eNOS in HUVECs treated with 50 *μ*g/ml AGEs (Figure 3(a)). These results suggested that profilin-1 requires the presence of AGEs to induce endothelial damage and ROS generation.

### 3.4. Involvement of RhoA/Rock1 in Profilin-1-Mediated Endothelial Injury

**Figure 4:**
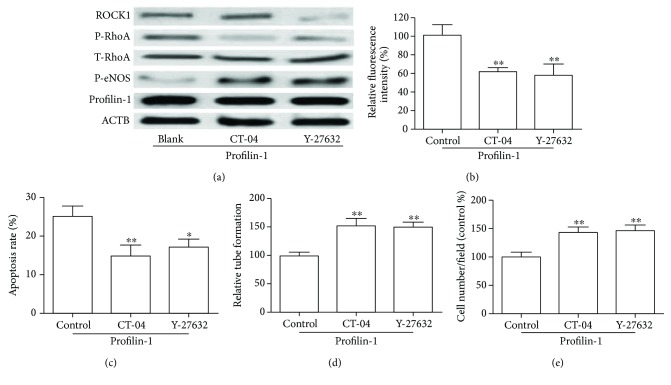
RhoA/ROCK1 activation mediated profilin-1-induced cell damage and ROS production. HUVECs stably overexpressing profilin-1 were treated with 5 *μ*g/ml CT-04 or 10 *μ*M Y-27632 for 24 h. (a) Expression of profilin-1, T-RhoA, p-RhoA, p-eNOS, and ROCK1 was determined by Western blot. (b) Intracellular ROS, (c) apoptosis, (d) tube formation, and (e) migratory abilities were analyzed. ^∗^*P* < 0.05; ^∗∗^*P* < 0.01.

RhoA/Rock1 is known to induce endothelial injury [21, 22]. To investigate whether RhoA/Rock1 is involved in profilin-1-mediated endothelial injury, we treated cells that stably overexpress profilin-1 with CT-04 (1 *μ*g/ml) or Y-27632 (10 *μ*M). As shown in Figure 4(a), CT-04 and Y-27632 were able to increase the expression of p-eNOS that was downregulated by profilin-1 (Figure 4(a)). Interestingly, this suppressive effect of profilin-1 was prevented when HUVECs were pretreated with CT-04 or Y-27632, which suggested the involvement of RhoA/ROCK1 in the profilin-1-mediated inhibitory effects (Figures 4(b)–4(e)).

### 3.5. ROS Were Required for Profilin-1 to Activate the RhoA/Rock1 Pathway in HUVECs

**Figure 5:**
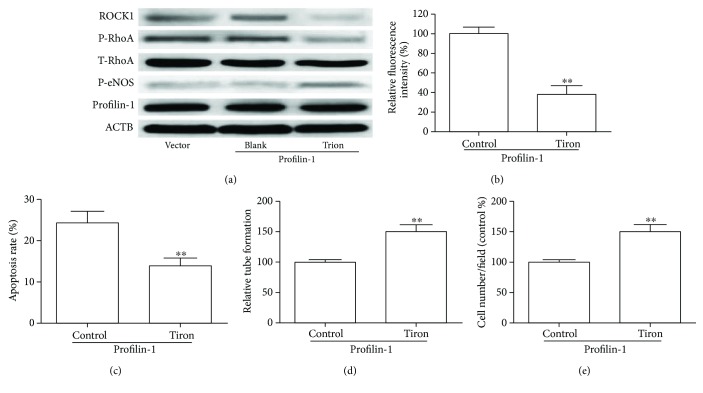
The effect of Tiron on profilin-1-mediated RhoA/ROCK1 activation in HUVECs. HUVECs stably overexpressing profilin-1 were treated with 10 *μ*M Tiron 12 h before application of 50 *μ*g/ml AGEs. (a) Western blot analysis of profilin-1, T-RhoA, p-RhoA, p-eNOS, and ROCK1 in profilin-1 overexpression HUVECs treated with Tiron under 50 *μ*g/ml AGEs. (b) Intracellular ROS, (c) apoptosis, (d) tube formation, and (e) migratory abilities were analyzed in profilin-1 overexpression HUVECs treated with Tiron under 50 *μ*g/ml AGEs. ^∗∗^*P* < 0.01

Previous reports have shown that ROS could activate RhoA/ROCK1 and cause endothelial cell injury [17, 23, 24]. Here, we determined whether ROS exerted their inhibitory effects on endothelial injury through the RhoA/ROCK1 signaling pathway. HUVECs stably overexpressing profilin-1 were exposed to AGEs (50 *μ*g/ml) for 24 h in the presence and absence of Tiron, an ROS scavenger. As shown in Figure 5(a), we observed that CT-04 prevented profilin-1-induced increases of p-RhoA and ROCK1 and decreases of p-eNOS. Moreover, profilin-1 significantly increased ROS production and Tiron (5 mM) prevented the increase of ROS in HUVECs exposed to AGEs (Figure 5(b)). The alteration of cell apoptosis, tube formation, and migration induced by profilin-1 was reverted by Tiron treatment (Figures 5(c)–5(e)). Taken together, these observations indicate that profilin-1 activated the RhoA/ROCK1 pathway through ROS.

## 4. Discussion

Numerous studies have suggested that diabetes is associated with various cardiovascular complications, which are the principal causes of morbidity and mortality of patients with diabetes [2, 3, 25]. Increasing amounts of evidence have indicated that larger amounts of ROS, produced by electron transport chain activation, result in oxidative stress and further aggravate the progression of diabetes and its complications [26, 27]. Profilin-1 has been found to regulate vascular remodeling via vascular inflammation and oxidative stress [11, 12, 14]. In the current study, we demonstrated that AGEs could induce profilin-1 expression and profilin-1 mediated endothelial damage and oxidative stress via the RhoA/ROCK1 signaling pathway and that the inhibition of profilin-1 attenuates RhoA/ROCK1 pathway activity and oxidative stress in HUVECs exposed to AGEs.

Previous studies have demonstrated that AGEs cause vascular dysfunction by interacting with their receptors and activating complex signaling pathways, resulting in the increased generation of ROS [14, 28, 29]. We investigated the influence of AGEs on profilin-1 in HUVECs to explore the underlying mechanisms of intracellular ROS production by AGEs. Our data showed that profilin-1 was significantly increased in HUVECs treated with AGEs and accompanied with release of ROS. These results indicated that AGEs have the ability to cause endothelial injury and increase the expression of profilin-1. In contrast, inhibition of profilin-1 with siRNA reversed AGE-induced ROS production and alteration of apoptosis. The RhoA/ROCK1 signaling pathway is known to play an important role in endothelial cell injury caused by hyperglycemia in vitro [30, 31]. In the present study, we found that AGE-induced RhoA and ROCK1 were markedly decreased after profilin-1 silencing. The above results revealed that profilin-1 was participating in the AGE-induced endothelial damage and ROS generation. Additionally, overexpression of profilin-1 promoted endothelial cell injury and ROS production, followed by the increase of p-RhoA and ROCK1 in HUVECs treated with 50 *μ*g/ml AGEs. These results suggest that profilin-1 requires the presence of AGEs to induce endothelial damage and ROS generation. Furthermore, profilin-1-mediated cell functions in HUVECs were partly blocked with the treatment of CT-04 or Y-27632. These findings provide evidence to support the fact that the RhoA/ROCK1 signaling pathway plays an important role in the generation of excess ROS by profilin-1 in HUVECs after exposure to extrinsic AGEs.

**Figure 6:**
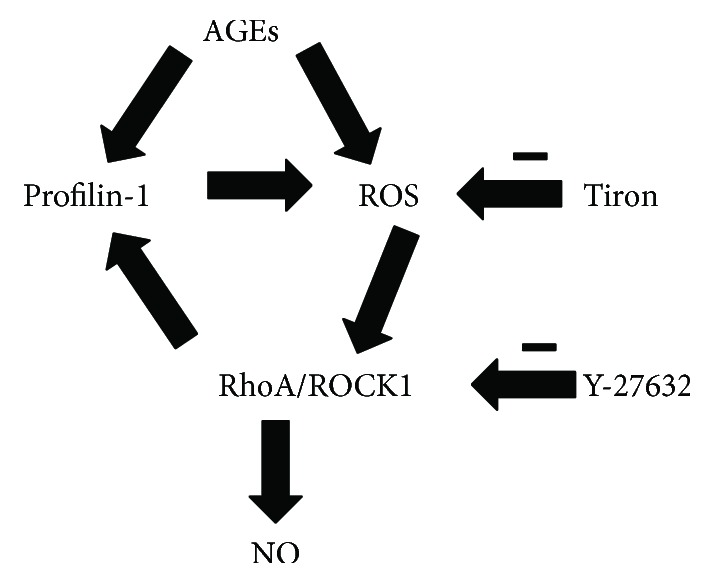
Proposed signaling pathway of the profilin-1 involved in endothelial damage and oxidative stress. AGEs upregulate the expression of profilin-1, activate RhoA/ROCK1 signaling, and stimulate the production of ROS. Treatment with ROS scavenger, RhoA/ROCK1 signaling inhibitor, or profilin-1-specific shRNA (sh-profilin-1) abolished the positive feedback loop.

It is well documented that increased ROS production contributes to endothelial injury in diabetes [3, 32]. Further investigation has shown that ROS could activate the Rho/ROCK1 signaling pathway, which is a key modulator of the actin cytoskeleton and plays an important role in oxidative stress [17, 31]. Whether ROS were involved in profilin-1-mediated Rho/ROCK1 signaling pathway was not known. Similar to the profilin-1 knockdown, we found that CT-04 prevented profilin-1-inducted ROS production and apoptosis and inhibited vascular-like tube formation and migration. These results indicated that ROS was required for profilin-1 activation of the Rho/ROCK signaling pathway. Shao et al. have shown that ROCK1 could mediate phosphorylation of profilin-1 at Ser-137 in neurodegeneration [33]. Thus, we concluded that increased profilin-1, induced by AGEs, resulted in ROS release and subsequently activated the Rho/ROCK1 signaling pathway (Figure 6). In conclusion, our data provide new molecular insights into the regulation of the endothelial dysfunction induced by AGEs and may provide novel therapeutic strategies for clinical treatment.
